# Trends and disparities in disseminated intravascular coagulation-related mortality among adults aged 25 and above in the U.S., 1999–2020: CDC WONDER insights

**DOI:** 10.1186/s12959-026-00848-7

**Published:** 2026-02-25

**Authors:** Muhammad Yousuf Saleem, Muhammad Salman Mustafa, Areeba Shafiq, Sameer Khan, Mudassir Rauf, Hasibullah Aminpoor, Muhammad Bilal Khan, Deep Birjani, Maryam Ahmed, Jazza Amir

**Affiliations:** 1 Department of Medicine and Surgery, Jinnah Sindh Medical University, Karachi, Pakistan; 2 Department of Medicine and Surgery, Dow University of Health Sciences, Karachi, Pakistan; 3 Faculty of Medicine, Kabul University of Medical Sciences “Abu Ali Ibn Sina”, Ata Turk Avenue, Jamal Mena, 3rd District, Kabul, 1001 Afghanistan

**Keywords:** Disseminated intravascular coagulation, Mortality, Epidemiology, Trends, CDC WONDER

## Abstract

**Background:**

Disseminated intravascular coagulation (DIC) is a severe, life-threatening coagulopathy often secondary to infection, malignancy, or obstetric complications. Despite advances in critical care, national patterns and disparities in DIC-related mortality remain underexplored.

**Objectives:**

To examine temporal, demographic, and regional trends in DIC-related mortality among U.S. adults from 1999 to 2020.

**Methods:**

Death certificate data were obtained from the CDC WONDER (Centers for Disease Control and Prevention Wide-Ranging Online Data for Epidemiologic Research) database for adults aged ≥ 25 years with DIC listed as an underlying or contributing cause of death. Age-adjusted mortality rates (AAMRs) per 100,000 population and annual percent change (APC) were calculated using Joinpoint regression and stratified by sex, race/ethnicity, age group, urbanization, and region.

**Results:**

From 1999 to 2020, 71,241 DIC-related deaths were identified among U.S. adults. The overall AAMR declined from 2.1 per 100,000 in 1999 to 1.6 in 2020. Men had higher mortality than women (1.6 vs. 1.5), and non-Hispanic (NH) Black adults had the highest AAMR (2.8), followed by Hispanic (1.6), and NH White adults (1.4). Mortality was highest among adults ≥ 85 years (6.1), residents of nonmetropolitan areas (1.6), and those in the South (1.6) and Northeast regions (1.5).

**Conclusions:**

Following a prolonged decline, DIC-related mortality in U.S. adults has risen since 2017, particularly among men, Black adults, and those in the South and rural areas. Targeted prevention and equitable access to critical care are essential to curb this resurgence.

**Supplementary Information:**

The online version contains supplementary material available at 10.1186/s12959-026-00848-7.

## Introduction

Disseminated intravascular coagulation (DIC) is a rare but often fatal systemic coagulopathy that complicates several clinical conditions, including sepsis, trauma, malignancy, and obstetric emergencies [1]. Despite advances in critical care and early recognition, DIC continues to carry a substantial mortality burden, with case-fatality rates reported between 20% and 50% in hospitalized patients [2]. In the United States, population-based data from the early 2000s suggested an incidence of 20–30 cases per 100,000 person-years, with little improvement in mortality over time [3]. Additionally, DIC inflicts a noticeable economic burden due to prolonged hospitalizations, intensive care requirements, and high treatment costs [4]. The risk of developing DIC increases with age as well as the presence of comorbidities such as cancer, cardiovascular diseases, and systemic infections [5, 6]. Mortality rates are particularly high among older adults and individuals with sepsis-associated DIC, in whom the risk of death may double compared with other etiologies [7, 8]. Despite this burden, most prior studies have relied on intensive-care or single-center data, limiting generalizability to the broader U.S. population. Consequently, long-term national trends and demographic disparities in DIC-related mortality remain poorly characterized [2].

Understanding the demographic and geographic distribution of DIC-associated deaths is essential for identifying populations at greatest risk and guiding preventive and clinical strategies. Therefore, we sought to examine the national trends and demographic disparities in DIC-related mortality in the U.S. population from 1999 to 2020 using the data from the Centers for Disease Control and Prevention Wide Ranging Online Data for Epidemiologic Research (CDC WONDER).

## Methods

We analyzed U.S. death certificate data from the CDC WONDER Multiple Cause-of-Death database [9]. Mortality data were extracted for individuals aged 25 years or older. Disseminated Intravascular Coagulation (DIC) is defined as a systemic, thrombo-hemorrhagic disorder characterized by the widespread pathological activation of coagulation cascade(s) in the body, which leads to the intravascular formation of fibrin hence consequently consumption of coagulation factors and platelets. The primary outcome was deaths due to DIC defined as ICD-10 code D65 listed as a cause of death anywhere in the death certificate. This code has been used in prior population-based studies assessing DIC-related mortality and epidemiologic trends [10–12]. ICD-10 definitions were applied in accordance with international and U.S. coding standards [13]. Since CDC WONDER contains de-identified data, this study was exempt from the Institutional Review Board (IRB) approval and complies with Strengthening the Reporting of Observational Studies in Epidemiology (STROBE) guidelines.

### Stratification

Analyses were stratified by calendar year (1999–2020), sex (male, female), race, Non-Hispanic (NH) American Indian or Alaska Native; NH Asian or Pacific Islander; NH Black or African American; NH White), ethnicity (Hispanic or Latino; non-Hispanic or Latino), 2013 National Center for Health Statistics (NCHS) urban–rural classification (Metropolitan and Non-metropolitan), and US Census region (Northeast, Midwest, South, West). Race and ethnicity categories reflect those recorded on death certificates in accordance with Office of Management and Budget (OMB) standards (10). Place of death (medical facility, home, hospice, nursing home/long-term care) was obtained for descriptive reporting only.

### Statistical analysis

Age-adjusted mortality rates (AAMRs) were calculated per 100,000 population by the direct method, standardized to the 2000 U.S. standard population, with 95% confidence intervals. DIC-related mortality trends were examined for yearly changes, age, demographics, and geographical locations. Temporal trends in AAMR were evaluated using the Joinpoint Regression Program (v4.9.0.0, National Cancer Institute [14]. Log-linear segmented regression models estimated annual percent change (APC) and detected inflection points where slopes changed significantly. To categorize APCs as increasing or decreasing, we evaluated their statistical deviation from the null hypothesis of zero change. Statistical significance was determined using a two-tailed t-test with a threshold of *p* < 0.05. CDC WONDER suppresses cells with counts < 10; suppressed estimates were excluded or aggregated. The heat map/state map was generated using Datawrapper (Datawrapper GmbH, Berlin, Germany), while all other study figures and graphical illustrations were created using Canva (Canva Pty Ltd, Sydney, Australia).

## Results

A total of 71,241 DIC-related deaths were recorded between 1999 and 2020 among adults aged ≥ 25. Amongst them, 52.8% were females and 47.2% were males (Supplemental Table 1). Information on the location of death was available for 71,012. Out of which 94.8% occurred within medical facilities, 2% occurred at home, 1.5% occurred in nursing homes/long-term care facilities, and 0.9% occurred in hospices (Supplemental Table 2).

### Annual trend for DIC-related AAMR

**Fig. 1:**
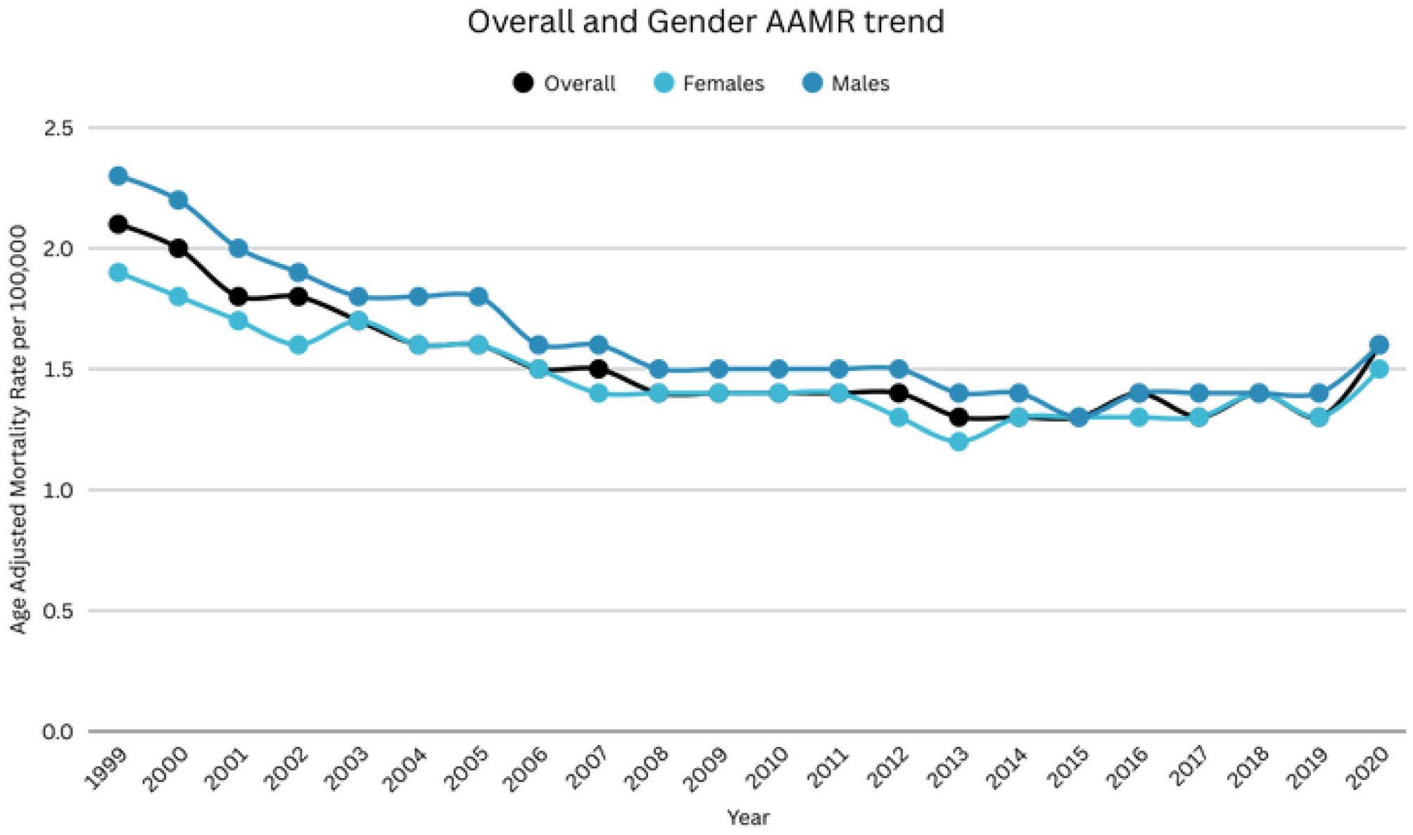
DIC-related mortality in the adult US population, 1999–2020, overall and gender stratified trend. Overall APC: 1999–2007: -4.71 (95% CI: -6.02 to -3.37), 2007–2017: -1.21 (95% CI: -2.24 to 0.17), 2017–2020: 5.89 (95% CI: -0.57 to 12.79). Female APC: 1999–2013: -2.73 (95% CI: -3.21 to -2.25), 2013–2020: 1.88 (95% CI: 0.25 to 3.54). Male APC: 1999–2003: -6.01 (95% CI: -8.52 to -3.42), 2003–2015: -2.48 (95% CI: -3.22 to -1.73), 2015–2020: 2.78 (95% CI: 0.03 to 5.60)

The AAMR for DIC-related deaths in adults was 2.1 in 1999 and 1.6 in 2020. Notably, the AAMR showed a significant decline from 2.1 in 1999 to 1.5 in 2007 (APC: -4.71; 95% CI: -6.02 to -3.37), which further declined till 1.3 in 2017 (APC: -1.21; 95% CI: -2.24 to -0.17). Subsequently, the AAMR showed a non-significant increase till 1.6 in 2020 (APC: 5.89; 95% CI: -0.57 to 12.79) (Supplemental Table 3, Fig. 1). Across the entire study period (1999–2020), the overall average annual percent change (AAPC) in DIC-related age-adjusted mortality − 1.42% (95% CI: −2.45 to − 0.38) indicated a significant long-term decline despite recent increases in mortality.

### DIC-related AAMR stratified by sex

Men had higher AAMRs than women throughout the study duration (overall AAMR men: 1.6; 95% CI: 1.6 to 1.6; women: 1.5; 95% CI: 1.5 to 1.5). The AAMR for males was 2.3 in 1999, which declined to 1.8 in 2003 (APC: -6.01; 95% CI: -8.52 to -3.42), this was followed by a further decrease to 1.3 in 2015 (APC: -2.48; 95% CI: -3.22 to -1.73), which then increased to 1.6 in 2020 (APC: 2.78; 95% CI: 0.03 to 5.60). Similarly, the AAMR for women was 1.9 in 1999, which decreased to 1.2 in 2013 (APC: -2.73, 95% CI: -3.21 to -2.25). After this, there was a slight rise till 2020 (APC: 1.88; 95% CI: 0.25 to 3.54) (Supplemental Tables 1, 3, Fig. 1). Over the entire study period (1999–2020), the overall AAPC demonstrated a significant long-term decline in DIC-related mortality for both sexes, with a steeper decrease observed among women (AAPC: −1.95%; 95% CI: −2.78 to − 1.10) compared with men (AAPC: −1.22%; 95% CI: −1.80 to − 0.64).

### DIC-related AAMR stratified by race/ethnicity

**Fig. 2:**
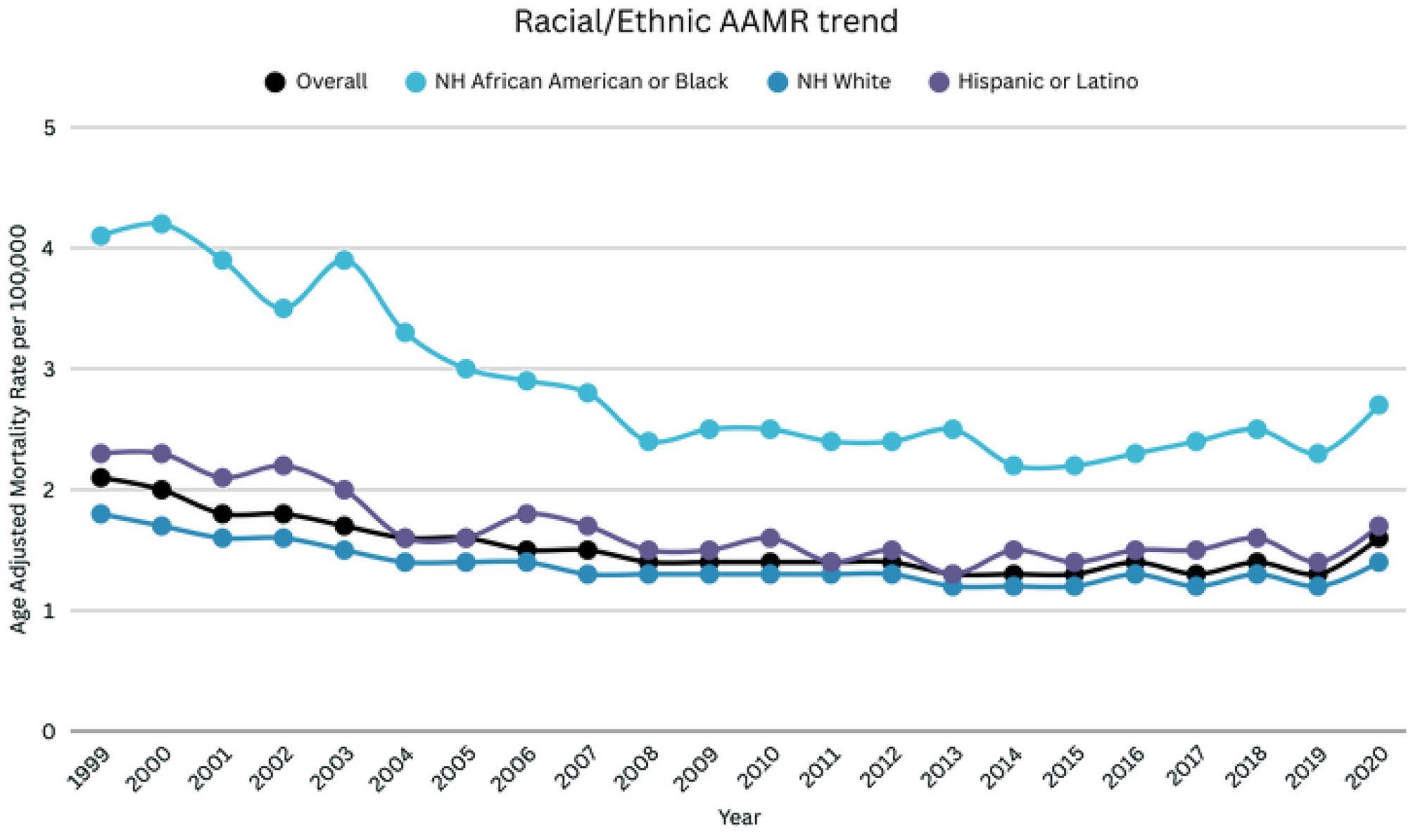
DIC-related mortality in the adult US population, 1999–2020, Race/Ethnicity trend. NH Black APC: 1999–2010: -5.56 (95% CI: -6.89 to -4.21), 2010–2020=: 0.52 (95% CI: -1.06 to 2.13). Hispanics APC: 1999–2011: -4.13 (95% CI: -5.40 to -2.81), 2011–2020=: 1.45 (95% CI: -0.70 to 3.65). NH Whites APC: 1999–2007: -3.93 (95% CI: -5.08 to -2.76), 2007–2020=: -0.12 (95% CI: -0.81 to 0.57)

When stratified by race/ethnicity, NH Blacks had the highest AAMR, followed by Hispanics or Latinos, and NH Whites (overall AAMR NH Blacks: 2.8; 95% CI: 2.8–2.9; Hispanics or Latinos: 1.6; 95% CI: 1.6–1.6; NH Whites: 1.3; 95% CI: 1.3–1.4). While analyzing trends for Blacks, there was a decline in mortality rate from 1999 to 2010 (APC: -5.56; 95% CI: -6.89 to -4.21), after which there was a period of stabilization till 2020 (APC: 0.52; -1.06 to 2.13). Similarly, the mortality rate in Hispanics or Latinos, also decreased from 1999 to 2011 (APC: -4.13; 95% CI: -5.4 to -2.81), which was followed by a non-significant rise in AAMR till 2020 (APC: 1.45; 95% CI: -0.70 to 3.65). Subsequently, for NH Whites, the mortality rate decreased from 1999 to 2007 (APC: -3.93; 95% CI: -5.08 to -2.76), which was followed by a non-significant decrease till 2020 (APC: -0.12; 95% CI: -0.81 to 0.57) (Supplemental Tables 1, 4, Fig. 2). From 1999 to 2020, DIC-related mortality declined significantly among Hispanic adults (AAPC − 2.71%; 95% CI − 3.67 to − 1.75), non-Hispanic Black adults (AAPC − 1.78%; 95% CI − 2.88 to − 0.67), and non-Hispanic White adults (AAPC − 1.59%; 95% CI − 2.17 to − 1.01).

### DIC-related AAMR stratified by age group

**Fig. 3:**
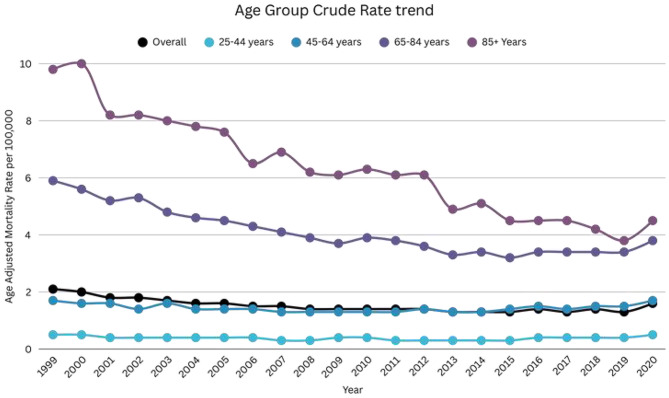
DIC-related mortality in the adult US population, 1999–2020, Age Group AAMR trend. 25–44 years APC: 1999–2014: -2.95 (95% CI: -4.22 to -1.67), 2014–2020: 8.13 (95% CI: 2.76 to 13.77). 45–64 years APC: 1999–2010: -2.37 (95% CI: -3.62 to -1.11), 2010–2020: 2.43 (95% CI: 1.34 to 3.52). 65–84 years APC: 1999–2014: -3.79 (95% CI: -4.18 to -3.40), 2014 to 2020: 2.04 (95% CI: -0.01 to 4.14). 85 + years APC: 1999 to 2020: -4.15 (95% CI: -4.58 to -3.72)

When stratifying the adults into 20-year age groups, the 85 + age group consistently displayed highest mortality, followed by 65–84 age group, 45–64 age group, 25–44 age group, (overall mean Crude rate 85+: 6.1; 95% CI: 6-6.2; 65–84: 4; 95% CI: 4-4.1; 45–64: 1.4; 95% CI: 1.4–1.5; 25–44: 0.4; 95% CI: 0.4–0.4). The mortality rate in the 85 + age group declined gradually from 1999 to 2020 (APC: -4.15; 95% CI: -4.58 to -3.72). In the 65–84 age group, the mortality rate decreased from 1999 to 2014 (APC: -3.79; 95% CI: -4.18 to -3.40), followed by a period of stabilization from 2014 to 2020 (APC: 2.04; 95% CI: -0.01 to 4.14). In patients aged 45–64, the mortality declined from 1999 to 2010 (APC: -2.37; 95% CI: -3.62 to -1.11), followed by an increase until 2020 (APC: 2.43; 95% CI: 1.34 to 3.52) In patients aged 25–44, the mortality rate decreased significantly from 1999 to 2014 (APC: -2.95; 95% CI: -4.22 to -1.67), followed by an increase till 2020 (APC: 8.13; 95% CI: 2.76 to 13.77) (Supplemental Table 8, Fig. 3). Over the 1999–2020 period, long-term trends in DIC-related mortality differed markedly by age. Mortality rates among adults aged 25–44 years (AAPC: 0.09%; 95% CI: −1.51 to 1.71) and 45–64 years (AAPC: −0.12%; 95% CI: −0.90 to 0.67) showed no statistically significant change. In contrast, significant declines were observed among adults aged 65–84 years (AAPC: −2.16%; 95% CI: −2.75 to − 1.57) and ≥ 85 years (AAPC: −4.16%; 95% CI: −4.58 to − 3.73), with the steepest reduction occurring in the oldest age group.

### DIC-related AAMR stratified by census region

Adults living in the South had the highest mortality rates, followed by the Northeast, the West, and the Midwest (overall AAMR South: 1.6; 95% CI: 1.6–1.7; Northeast: 1.5; 95% CI: 1.5–1.5; West: 1.4; 95% CI: 1.4–1.4; Midwest: 1.3; 95% CI: 1.3–1.4).

**Fig. 4:**
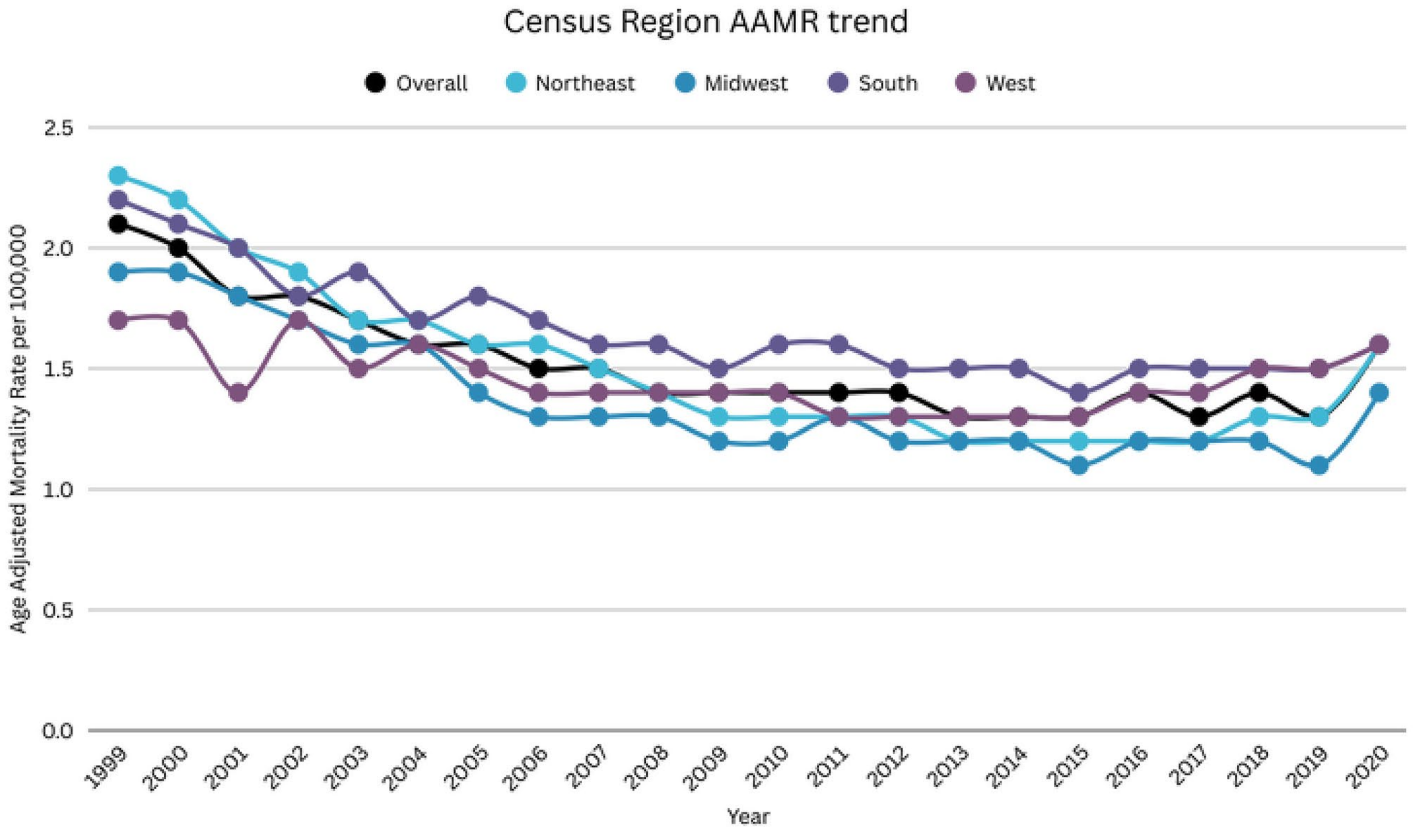
DIC-related mortality in the adult US population, 1999–2020, Census Region AAMR trend. South APC: 1999–2009: -3.37 (95% CI: -4.15 to -2.58), 2009–2020: -0.05 (95% CI: -0.88 to 0.78). Northeast APC: 1999–2009: -5.30 (95% CI: -5.90 to -4.61), 2009–2017: -1.41 (95% CI: -3.16 to -0.37), 2017–2020: 9.73 (95% CI: 3.51 to 16.31). West APC: 1999–2014: -1.85 (95% CI: -2.42 to -1.27), 2014–2020: 3.89 (95% CI: 1.43 to 6.39). Midwest APC: 1999–2009: -4.82 (95% CI: -5.87 to -3.75), 2009–2020: 0.31 (95% CI: -0.87 to 1.52), 2009–2018: -1.20 (95% CI: -2.16 to -0.24)

Adults living in the South region showed a decrease in mortality rate from 1999 to 2009 (APC: -3.37; 95% CI: -4.15 to -2.58), followed by a brief period of stability until 2020 (APC: -0.05; 95% CI: -0.88 to 0.78). Similarly, Northeast region showed a decrease in mortality rate from 1999 to 2020 (APC: -5.30; 95% CI: -5.9 to -4.61), a non-significant decline until 2017 (APC: -1.41; 95% CI: -3.16 to 0.37), and an increase from 2017 to 2020 (APC: 9.73; 95% CI: 3.51 to 16.31). Adults living in the West showed a steep decline from 1999 to 2014 (APC: -1.85; 95% CI: -2.42 to -1.27). However, this was followed by an increase till 2020 (APC: 3.89; 95% CI: 1.43 to 6.39). The Midwest region showed a notable decline till 2009 (APC: -4.82; 95% CI: -5.87 to -3.75), followed by a period of stabilization until 2020 (APC: 0.31; 95% CI: -0.87 to 1.52) (Supplemental Table 6, Fig. 4). Over the entire study period (1999–2020), long-term trends in DIC-related mortality differed by census region. Significant overall declines were observed in the Northeast (AAPC: −2.17%; 95% CI: −2.91 to − 1.42), South (AAPC: −1.80%; 95% CI: −2.81 to − 0.77), and West (AAPC: −1.65%; 95% CI: −2.18 to − 1.11). In contrast, mortality rates in the Midwest remained relatively stable over time, with no statistically significant overall change (AAPC: −0.24%; 95% CI: −0.98 to 0.50).

### DIC-related AAMR stratified by 2013 urbanization level

**Fig. 5:**
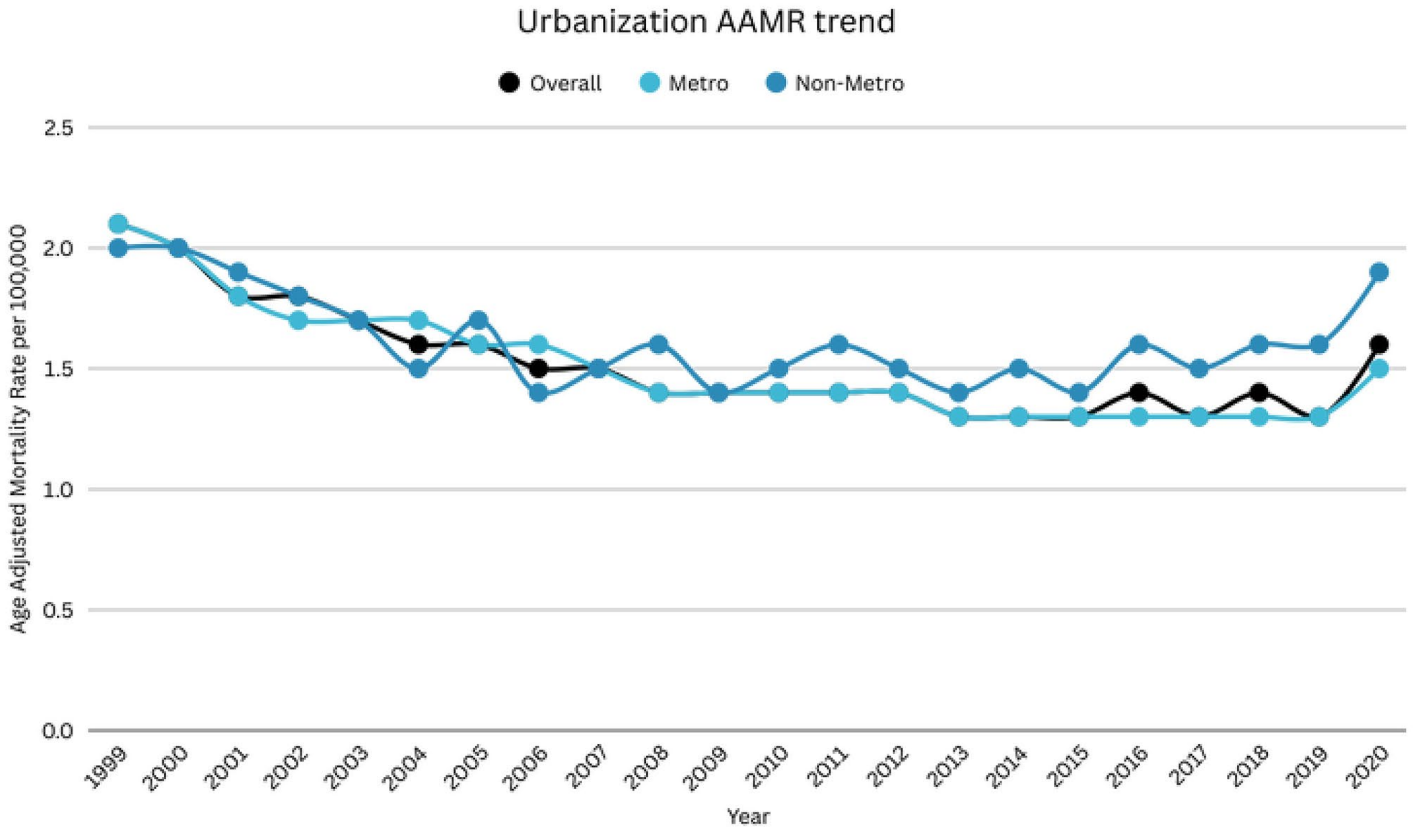
DIC-related mortality in the adult US population, 1999–2020, Urbanization AAMR trend Nonmetropolitan APC: 1999–2009: -3.45 (95% CI: -4.89 to -1.98), 2009–2020: 1.71 (95% CI: 0.30 to 3.14). Metropolitan APC: 1999–2001: -7.49 (95% CI: -12.77 to -1.89), 2001–2009=: -3.08 (95% CI: -4.07 to -2.08), 2009–2018: -1.20 (95% CI: -2.16 to -0.24)

A significant variation in AAMR was observed when comparing metropolitan and nonmetropolitan areas. Nonmetropolitan areas consistently had higher AAMR than metropolitan areas throughout the study period (overall AAMR Nonmetropolitan: 1.6; 95% CI: 1.6–1.6; Metropolitan: 1.5; 95% CI: 1.5–1.5). The mortality rate for nonmetropolitan areas decreased from 1999 to 2009 (APC: -3.45; 95% CI: -4.89 to -1.98), followed by a significant increase till 2020 (APC: 1.71; 95% CI: 0.30 to 3.14). AAMR of metropolitan areas declined from 1999 to 2001 (APC: -7.49; 95% CI: -12.77 to -1.89), followed by a further decrease till 2009 (APC: -3.08; 95% CI: -4.07 to -2.08). Afterward, it again decreased until 2018 (APC: -1.20; 95% CI: -2.16 to -0.24), leading to a rise till 2020 (APC: 7.88; 95% CI: -1.06 to 17.63) (Supplemental Table 5, Fig. 5). Over the entire study period (1999–2020), nonmetropolitan areas experienced a significant overall decline in DIC-related mortality (AAPC: −1.72%; 95% CI: −2.72 to − 0.71). In contrast, metropolitan areas showed a smaller, non-significant overall decline (AAPC: −0.78%; 95% CI: −1.72 to 0.17).

### DIC-related AAMR stratified by state

**Fig. 6:**
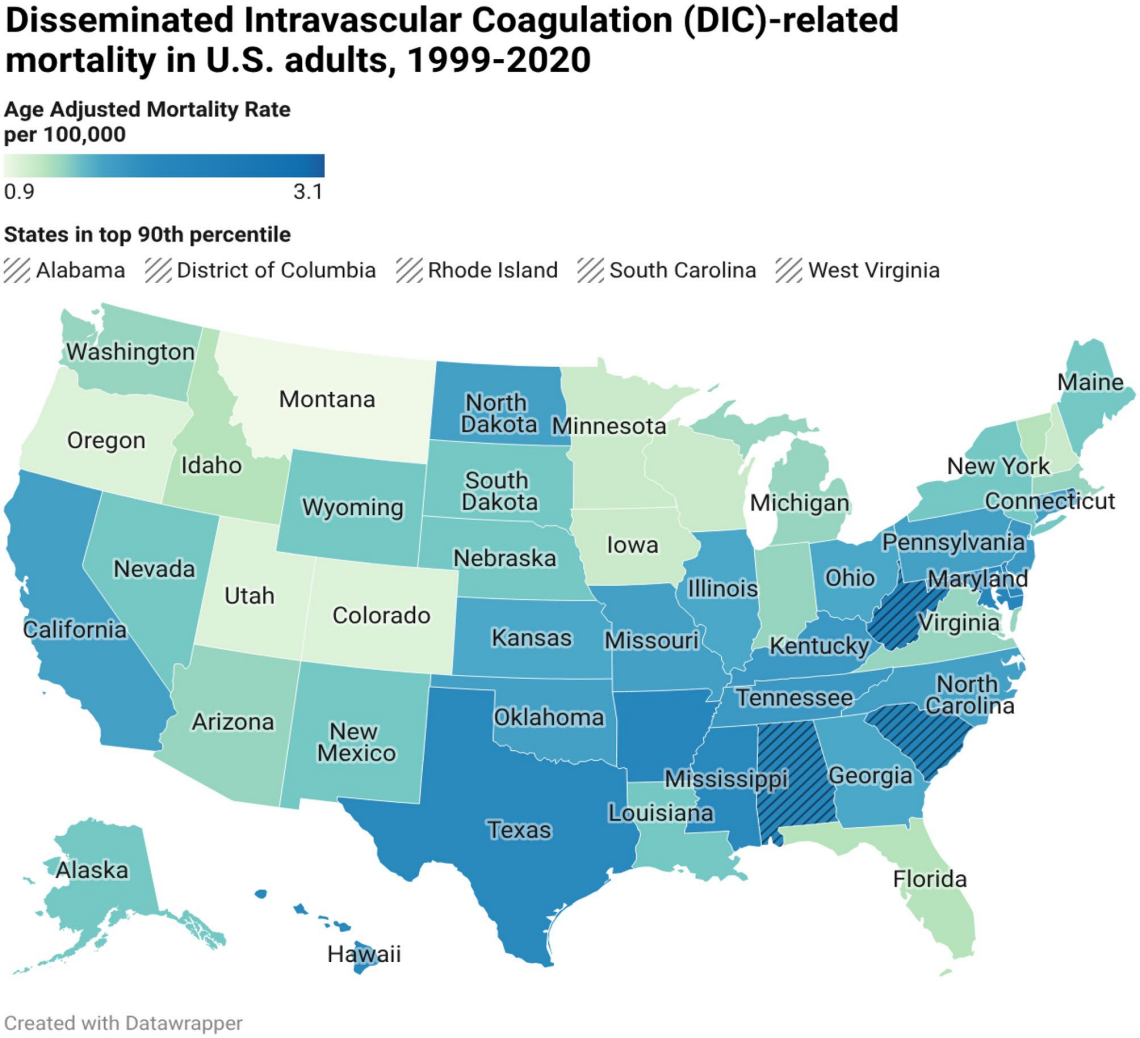
DIC-related mortality in the adult US population, 1999–2020, stratified by state

A considerable variation in AAMR was observed in different states, from 0.9 (95% CI: 0.7-1) in Montana to 3.1 (95% CI: 2.7–3.4) in the District of Columbia. States that fell in the top 90th percentile were the District of Columbia, West Virginia, Rhode Island, South Carolina, and Alabama, which had approximately double the AAMR as compared to the states that fell in the lower 10th percentile, which include Wisconsin, Colorado, Oregon, Utah, and Montana (Supplemental Table 7, Fig. 6).

## Discussions

In this analysis of U.S. mortality data from 1999 to 2020, Disseminated Intravascular Coagulation (DIC) demonstrated a persistent but evolving public health burden. Overall, DIC-related age-adjusted mortality rates declined substantially in the early 2000s, plateaued in the mid-2010s, and reversed upward toward 2020. Despite long-term improvement, mortality remained disproportionately high among older adults, men, residents of the South, rural populations, and non-Hispanic Black individuals. Geographic and demographic disparities persisted throughout the study period, underscoring the combined influence of population aging, uneven access to critical care, and differential exposure to sepsis, malignancy, and COVID-19–related coagulopathy.

Over the study period, DIC-related age-adjusted mortality exhibited a multi-phase pattern, with early declines followed by attenuation and a late-series reversal. The initial reduction in the early 2000s is consistent with improvements in critical care and the progressive implementation of standardized sepsis recognition and treatment bundles [15] which were associated with declining sepsis case-fatality even as sepsis ascertainment increased in administrative and clinical datasets [16], since sepsis is plausibly the leading cause of DIC in the US population. After early improvements, mortality declines slowed in the mid-2010s, a plausible explanation is etiologic and diagnostic heterogeneity across age bands and time. DIC arises not from a fixed, but multiple triggers (sepsis, cancer, trauma, obstetric etiologies), and its clinical identification depends on evolving scoring frameworks and thresholds (e.g., ISTH overt DIC and related criteria), which can shift the case-mix captured on death certificates over time due to changes in how DIC is recognized and documented, which together may alter the profile of deaths attributed to DIC across years. Finally, the late upturn near the end of the series is biologically plausible in the context of COVID-19–associated coagulopathy [17] and pandemic-era care disruption [18], both of which were temporally aligned with increased thrombo-inflammatory complications and excess mortality in the U.S. Together, these findings suggest that temporal changes in DIC-related mortality reflect evolving clinical contexts and population vulnerability, rather than uniform improvement or deterioration in DIC-specific care.

Although DIC-related mortality declined in both sexes early in the study period, males consistently experienced higher mortality, with less sustained improvement over time. This pattern suggests that men may more often develop DIC in the setting of severe or less reversible illness, limiting the degree to which advances in care translate into continued mortality reductions. Men are more likely to encounter upstream conditions that precipitate DIC, including severe infections and trauma or chronic diseases like diabetes or hyperlipidemia [19], increasing the likelihood of entering the DIC pathway in the setting of advanced or rapidly progressive illness, such as advanced sepsis [21, 20] or trauma, which limits the degree to which advances in care translate into continued mortality reductions once DIC develops [22]. This pattern likely also reflects a combination of clinical and biological factors. In addition, sex-based differences in immune and coagulation responses may modestly influence outcomes. Experimental and clinical studies suggest that estrogen may exert endothelial-protective and anti-inflammatory effects [23], in contrast, male-predominant hormonal and immune profiles may be associated with greater inflammatory and coagulopathic responses during critical illness. While these biological differences are unlikely to fully explain the observed disparity, they may contribute to higher fatality once DIC is established, reinforcing the persistently elevated mortality observed among males.

Marked racial disparities were observed, with NH Black adults experiencing substantially higher DIC-related mortality than other groups. This disparity aligns with broader U.S. evidence showing persistent Black–White gaps in life expectancy [24], driven by higher mortality from multiple causes including cardiovascular disease [25], diabetes [26], endocrine disorders, cancer, and injury, indicating a cumulative burden of chronic and acute disease rather than a single-pathway effect. Because DIC typically develops as a late manifestation of critical illness, greater severity at presentation substantially increases fatality once DIC occurs. The early decline across all races in DIC mortality likely reflects improvements in upstream conditions that historically carried disproportionate burden in this population, including reductions in HIV-related mortality following widespread antiretroviral therapy [27], improvements in sepsis recognition, and advances in obstetric emergency care [28]. However, the persistence of a large mortality gap despite these early gains specially in NH Black individuals, suggests that later-stage determinants particularly delayed escalation to intensive care and care delivery within resource-constrained hospital settings continue to amplify fatality once DIC develops. Thus, the pronounced and persistent NH Black excess in DIC-related mortality likely reflects a convergence of higher disease severity at onset and unequal capacity to reverse critical illness once DIC is established, rather than differences in disease recognition alone. While causal inference is limited by the ecological nature of death-certificate data, the magnitude and consistency of this disparity underscores the need for equity-focused improvements in early illness detection, rapid escalation pathways, and critical care delivery in populations at highest risk.

By age, DIC mortality followed a steep gradient, with the oldest adults experiencing the highest AAMR. This pattern is expected because the major clinical contexts in which DIC occurs, severe infection/sepsis, advanced malignancy, and multi-organ failure, are concentrated in older populations and carry higher short-term fatality with advancing age. However, age-stratified temporal patterns were not uniform. While the oldest age groups experienced sustained declines in DIC-related mortality, several younger and midlife strata exhibited attenuation or late-period increases. These differences likely reflect age-specific clinical pathways leading to DIC rather than uniform effects across the lifespan. As mentioned, in older adults, DIC most commonly arises in the setting of sepsis, advanced malignancy, or end-stage organ failure [29]. In contrast, DIC occurring in midlife adults is more often precipitated by abrupt, high-severity events such as severe infection, trauma, or treatment-related complications [30], which may present later in the disease course and progress rapidly. Once DIC develops in these settings, outcomes are driven primarily by illness severity and timeliness of escalation rather than age itself [31]. Accordingly, late increases in DIC-related mortality among midlife adults likely reflect changes in disease severity, underlying etiologies, and access to timely care, rather than demographic ageing alone [32]. These findings underscore that temporal changes in DIC mortality are shaped by evolving clinical contexts and patterns of critical illness, not simply by population age structure.

Across U.S. census regions, DIC-related mortality declined during the early years of the study but subsequently diverged. While the Northeast and West demonstrated more sustained improvements, the South experienced an early decline followed by prolonged stagnation, remaining the region with the highest age-adjusted mortality throughout the study period.

The South overlaps substantially with multiple chronic disease ‘belts’ in the United States, including the diabetes [33, 34] and cardiovascular [35] disease belts, where higher prevalence of metabolic disease and infection-related complications may further amplify susceptibility to severe sepsis [36] and downstream coagulopathy, contributing to persistently elevated DIC-related mortality. However, these patterns may reflect differences in healthcare access and service delivery rather than differences in DIC biology alone. Delayed access to care due to lower insurance coverage [37] and greater reliance on emergency services may result in patients presenting with more advanced illness, increasing the likelihood of severe coagulopathy and fatal DIC. Geographic factors may further contribute, as larger rural populations and longer distances to tertiary centers can delay escalation to intensive care once DIC develops. In addition, variability in hospital resources and critical care capacity may limit the ability to monitor and manage complex coagulopathies rapidly. While early improvements in sepsis recognition and critical care likely drove initial mortality declines nationwide, uneven access to high-acuity care may have constrained further gains in the South, contributing to persistently elevated DIC mortality. Policy-level factors likely contributed to the persistence of these disparities. Medicaid expansion [38] under the Affordable Care Act was adopted earlier and more comprehensively in many North-eastern and Western states, whereas several Southern states expanded later or not at all. These coverage gaps have been associated with delayed care and more severe illness at presentation, limiting the durability of mortality reductions [39]. In addition, rural hospital closures [40] and uneven critical care capacity are more prevalent in parts of the South, potentially constraining the effectiveness of national sepsis quality initiatives and contributing to regional plateaus in DIC-related mortality.

DIC-related mortality also differed between urban and rural settings, with rural areas demonstrating less sustained improvement over time. These differences in mortality likely reflect both differences in exposure to severe illness and policy-driven differences in healthcare delivery capacity [41]. Rural populations are more likely to experience delayed presentation for acute infections, increasing the likelihood of progressing to advanced disease states that precipitate DIC. These risks are compounded by structural changes in the U.S. healthcare system, including rural hospital closures [42] and consolidation of high-acuity services, which have reduced local access to ICU-capable care and lengthened transfer pathways [43]. In addition, variability in Medicaid expansion and insurance coverage has disproportionately affected rural communities, contributing to delayed care-seeking and greater illness severity at presentation [44]. While national sepsis quality initiatives have improved outcomes overall, their sustained impact depends on timely escalation and resource availability, which remain uneven across urban and rural settings. Together, these system- and policy-level factors provide a plausible explanation for the early mortality declines followed by prolonged stagnation observed in rural areas.

State-level DIC mortality demonstrated clear geographic clustering, suggesting systematic rather than random variation [45]. Differences in Medicaid expansion status and insurance coverage [46], higher rates of rural hospital closures [47] and service contraction in non-expansion settings, and uneven ICU bed supply provide plausible explanations for why some states exhibit persistently elevated DIC mortality, particularly given the condition’s sensitivity to timely escalation and high-acuity support. Variation in the implementation and effectiveness of national sepsis quality initiatives, including SEP-1 [48], may further contribute to uneven downstream improvements in severe coagulopathy-related mortality across states.

Most DIC-attributed deaths occurred in hospital settings, which is expected given that DIC typically develops as a late complication of severe illness requiring inpatient and often intensive care. In contrast, deaths occurring outside the hospital may reflect rapid clinical deterioration or delays along the care pathway, including late presentation or delayed escalation to higher-acuity care after initial medical contact. Because conditions that precipitate DIC, particularly severe sepsis, can progress quickly, even short delays in escalation may substantially increase the likelihood of fatal outcomes.

## Limitations

This study has several limitations. It relies on retrospective national mortality data, which may be affected by coding errors, misclassification, or underreporting of disseminated intravascular coagulation (DIC) as a cause of death. Detailed patient-level clinical information, including comorbidities, illness severity, laboratory findings, and treatment interventions, was not available, limiting adjustment for potential confounding factors. While analyses examined trends and disparities by sex, age, race, state, and urbanization, other factors, such as socioeconomic status, healthcare access, and geographic differences beyond state-level variation, were not assessed and may influence observed patterns. Temporal changes in diagnostic practices, ICD coding, and medical management over the 1999–2020 study period could also have affected mortality trends. Non-Hispanic Asian and American Indian/Alaska Native populations were excluded from subgroup analyses because of small annual death counts and unstable age-adjusted mortality estimates in CDC WONDER, which limited the reliability of trend estimation; this exclusion represents an important limitation and may mask heterogeneity in DIC-related mortality among these groups. Additionally, focusing exclusively on deaths excludes information on DIC incidence, morbidity, and long-term outcomes. Finally, the use of aggregated population-level data precludes causal inferences at the individual level.

## Conclusion

After a significant decline in AAMR from 1999 to 2006, mortality stabilized until 2017, then rose again through 2020. The highest AAMR was observed in men, NH Blacks, nonmetropolitan areas, the South region, the District of Columbia, and in adults aged 85+. Overall mortality has improved since 1999, but a recent spike in AAMR, especially in middle-aged adults, nonmetropolitan residents, and racial, ethnic minorities, highlights widening disparities in access to advanced care and compounded effects of systemic vulnerabilities. Targeted prevention, timely recognition, and equitable access—particularly in rural and minority communities—are essential to further reduce DIC-related mortality.

## Supplementary Information

Below is the link to the electronic supplementary material.

Supplementary Material 1

## Data Availability

The data that support the findings of this study are available in CDC WONDER at https://wonder.cdc.gov/mcd.html. These data were derived from the following resources available in the public domain:- Multiple Cause of Death, 1999-2020, https://wonder.cdc.gov/mcd-icd10.html.
