# Supplementary material for: Trends and disparities in disseminated intravascular coagulation-related mortality among adults aged 25 and above in the U.S., 1999–2020: CDC WONDER insights
Source: Thromb J. 2026 Feb 25;24:26. doi: 10.1186/s12959-026-00848-7 (PMC12937566; doi:10.1186/s12959-026-00848-7)
Supplement: Supplementary file 1 — Supplementary Material 1 [file 12959_2026_848_MOESM1_ESM.docx]

**Supplemental Files:**

**Supplemental Table 1. Disseminated Intravascular Coagulation related mortalities in Adults in the United States, 1999 to 2020, demographics table**

| **Year** | **Overall** | **Women** | **Men** | **NH Whites** | **Hispanic** | **NH Blacks** | **Population** |
| --- | --- | --- | --- | --- | --- | --- | --- |
| **1999** | 3664 | 1912 | 1752 | 2811 | 246 | 708 | 180408769 |
| **2000** | 3543 | 1856 | 1687 | 2682 | 267 | 709 | 181984640 |
| **2001** | 3358 | 1757 | 1601 | 2543 | 243 | 691 | 184305128 |
| **2002** | 3255 | 1712 | 1543 | 2524 | 269 | 617 | 186208028 |
| **2003** | 3254 | 1765 | 1489 | 2412 | 264 | 714 | 188090429 |
| **2004** | 3138 | 1666 | 1472 | 2390 | 217 | 624 | 190205384 |
| **2005** | 3178 | 1679 | 1499 | 2447 | 240 | 592 | 192551384 |
| **2006** | 3030 | 1644 | 1386 | 2301 | 282 | 584 | 195019359 |
| **2007** | 2963 | 1574 | 1389 | 2267 | 281 | 559 | 197403777 |
| **2008** | 2950 | 1598 | 1352 | 2266 | 258 | 527 | 199795090 |
| **2009** | 2959 | 1571 | 1388 | 2259 | 283 | 544 | 202107016 |
| **2010** | 3060 | 1599 | 1461 | 2332 | 281 | 561 | 203891983 |
| **2011** | 3087 | 1649 | 1438 | 2389 | 285 | 546 | 206592936 |
| **2012** | 3111 | 1639 | 1472 | 2374 | 302 | 563 | 208826037 |
| **2013** | 2983 | 1517 | 1466 | 2229 | 289 | 590 | 211085314 |
| **2014** | 3084 | 1627 | 1457 | 2359 | 346 | 551 | 213809280 |
| **2015** | 3073 | 1636 | 1437 | 2317 | 337 | 578 | 216553817 |
| **2016** | 3329 | 1744 | 1585 | 2503 | 366 | 616 | 218641417 |
| **2017** | 3325 | 1738 | 1587 | 2491 | 404 | 660 | 221447331 |
| **2018** | 3481 | 1848 | 1633 | 2584 | 437 | 691 | 223311190 |
| **2019** | 3426 | 1817 | 1609 | 2532 | 392 | 684 | 224981167 |
| **2020** | 3990 | 2082 | 1908 | 2933 | 525 | 797 | 226635013 |
| **Total** | 71241 | 37630 | 33611 | 53945 | 6814 | 13706 | 4473854489 |

**Supplemental Table 2. Disseminated Intravascular Coagulation related mortalities in Adults in the United States, 1999 to 2020 stratified by Place of Death**

| **Place of Death** | **Deaths** | **% of Total Deaths** |
| --- | --- | --- |
| Medical Facility - Inpatient | 65409 | 91.80% |
| Medical Facility - Outpatient or ER | 1963 | 2.80% |
| Medical Facility - Dead on Arrival | 46 | 0.10% |
| Medical Facility - Status unknown | 105 | 0.10% |
| Decedent's home | 1391 | 2.00% |
| Hospice facility | 616 | 0.90% |
| Nursing home/long term care | 1098 | 1.50% |
| Other | 384 | 0.50% |
| Place of death unknown | 229 | 0.30% |

**Supplemental Table 3. Disseminated Intravascular Coagulation related mortalities in Adults in the United States, 1999 to 2020 stratified by Sex and year**

| **Variable** | **Year** | **Age Adjusted Rate** | **Age Adjusted Rate Lower 95% Confidence Interval** | **Age Adjusted Rate Upper 95% Confidence Interval** |
| --- | --- | --- | --- | --- |
| Overall | 1999 | 2.1 | 2 | 2.1 |
| Overall | 2000 | 2 | 1.9 | 2 |
| Overall | 2001 | 1.8 | 1.8 | 1.9 |
| Overall | 2002 | 1.8 | 1.7 | 1.8 |
| Overall | 2003 | 1.7 | 1.7 | 1.8 |
| Overall | 2004 | 1.6 | 1.6 | 1.7 |
| Overall | 2005 | 1.6 | 1.6 | 1.7 |
| Overall | 2006 | 1.5 | 1.5 | 1.6 |
| Overall | 2007 | 1.5 | 1.4 | 1.5 |
| Overall | 2008 | 1.4 | 1.4 | 1.5 |
| Overall | 2009 | 1.4 | 1.4 | 1.5 |
| Overall | 2010 | 1.4 | 1.4 | 1.5 |
| Overall | 2011 | 1.4 | 1.4 | 1.5 |
| Overall | 2012 | 1.4 | 1.3 | 1.4 |
| Overall | 2013 | 1.3 | 1.2 | 1.3 |
| Overall | 2014 | 1.3 | 1.3 | 1.4 |
| Overall | 2015 | 1.3 | 1.2 | 1.3 |
| Overall | 2016 | 1.4 | 1.3 | 1.4 |
| Overall | 2017 | 1.3 | 1.3 | 1.4 |
| Overall | 2018 | 1.4 | 1.4 | 1.5 |
| Overall | 2019 | 1.3 | 1.3 | 1.4 |
| Overall | 2020 | 1.6 | 1.5 | 1.6 |
| Female | 1999 | 1.9 | 1.8 | 2 |
| Female | 2000 | 1.8 | 1.8 | 1.9 |
| Female | 2001 | 1.7 | 1.6 | 1.8 |
| Female | 2002 | 1.6 | 1.6 | 1.7 |
| Female | 2003 | 1.7 | 1.6 | 1.8 |
| Female | 2004 | 1.6 | 1.5 | 1.6 |
| Female | 2005 | 1.6 | 1.5 | 1.6 |
| Female | 2006 | 1.5 | 1.4 | 1.5 |
| Female | 2007 | 1.4 | 1.3 | 1.5 |
| Female | 2008 | 1.4 | 1.3 | 1.5 |
| Female | 2009 | 1.4 | 1.3 | 1.4 |
| Female | 2010 | 1.4 | 1.3 | 1.4 |
| Female | 2011 | 1.4 | 1.3 | 1.5 |
| Female | 2012 | 1.3 | 1.3 | 1.4 |
| Female | 2013 | 1.2 | 1.2 | 1.3 |
| Female | 2014 | 1.3 | 1.2 | 1.4 |
| Female | 2015 | 1.3 | 1.2 | 1.4 |
| Female | 2016 | 1.3 | 1.3 | 1.4 |
| Female | 2017 | 1.3 | 1.2 | 1.4 |
| Female | 2018 | 1.4 | 1.3 | 1.4 |
| Female | 2019 | 1.3 | 1.3 | 1.4 |
| Female | 2020 | 1.5 | 1.5 | 1.6 |
| Male | 1999 | 2.3 | 2.2 | 2.4 |
| Male | 2000 | 2.2 | 2.1 | 2.3 |
| Male | 2001 | 2 | 1.9 | 2.1 |
| Male | 2002 | 1.9 | 1.8 | 2 |
| Male | 2003 | 1.8 | 1.7 | 1.9 |
| Male | 2004 | 1.8 | 1.7 | 1.9 |
| Male | 2005 | 1.8 | 1.7 | 1.8 |
| Male | 2006 | 1.6 | 1.5 | 1.7 |
| Male | 2007 | 1.6 | 1.5 | 1.7 |
| Male | 2008 | 1.5 | 1.4 | 1.5 |
| Male | 2009 | 1.5 | 1.4 | 1.6 |
| Male | 2010 | 1.5 | 1.5 | 1.6 |
| Male | 2011 | 1.5 | 1.4 | 1.5 |
| Male | 2012 | 1.5 | 1.4 | 1.5 |
| Male | 2013 | 1.4 | 1.3 | 1.5 |
| Male | 2014 | 1.4 | 1.3 | 1.4 |
| Male | 2015 | 1.3 | 1.3 | 1.4 |
| Male | 2016 | 1.4 | 1.4 | 1.5 |
| Male | 2017 | 1.4 | 1.3 | 1.5 |
| Male | 2018 | 1.4 | 1.3 | 1.5 |
| Male | 2019 | 1.4 | 1.3 | 1.4 |
| Male | 2020 | 1.6 | 1.5 | 1.7 |

**Supplemental Table 4. Disseminated Intravascular Coagulation related mortalities in Adults in the United States, 1999 to 2020 stratified by Race/Ethnicity.**

| **Hispanic Origin** | **Year** | **Age Adjusted Rate** | **Age Adjusted Rate Lower 95% Confidence Interval** | **Age Adjusted Rate Upper 95% Confidence Interval** |
| --- | --- | --- | --- | --- |
| Hispanic or Latino | 1999 | 2.3 | 2 | 2.6 |
| Hispanic or Latino | 2000 | 2.3 | 2 | 2.6 |
| Hispanic or Latino | 2001 | 2.1 | 1.8 | 2.4 |
| Hispanic or Latino | 2002 | 2.2 | 1.9 | 2.5 |
| Hispanic or Latino | 2003 | 2 | 1.7 | 2.2 |
| Hispanic or Latino | 2004 | 1.6 | 1.4 | 1.8 |
| Hispanic or Latino | 2005 | 1.6 | 1.4 | 1.8 |
| Hispanic or Latino | 2006 | 1.8 | 1.6 | 2 |
| Hispanic or Latino | 2007 | 1.7 | 1.5 | 1.9 |
| Hispanic or Latino | 2008 | 1.5 | 1.3 | 1.7 |
| Hispanic or Latino | 2009 | 1.5 | 1.3 | 1.7 |
| Hispanic or Latino | 2010 | 1.6 | 1.4 | 1.7 |
| Hispanic or Latino | 2011 | 1.4 | 1.3 | 1.6 |
| Hispanic or Latino | 2012 | 1.5 | 1.3 | 1.7 |
| Hispanic or Latino | 2013 | 1.3 | 1.2 | 1.5 |
| Hispanic or Latino | 2014 | 1.5 | 1.3 | 1.6 |
| Hispanic or Latino | 2015 | 1.4 | 1.2 | 1.5 |
| Hispanic or Latino | 2016 | 1.5 | 1.3 | 1.6 |
| Hispanic or Latino | 2017 | 1.5 | 1.3 | 1.6 |
| Hispanic or Latino | 2018 | 1.6 | 1.4 | 1.7 |
| Hispanic or Latino | 2019 | 1.4 | 1.2 | 1.5 |
| Hispanic or Latino | 2020 | 1.7 | 1.6 | 1.9 |
| Black or African American | 1999 | 4.1 | 3.8 | 4.5 |
| Black or African American | 2000 | 4.2 | 3.9 | 4.5 |
| Black or African American | 2001 | 3.9 | 3.6 | 4.2 |
| Black or African American | 2002 | 3.5 | 3.2 | 3.8 |
| Black or African American | 2003 | 3.9 | 3.6 | 4.2 |
| Black or African American | 2004 | 3.3 | 3 | 3.6 |
| Black or African American | 2005 | 3 | 2.8 | 3.3 |
| Black or African American | 2006 | 2.9 | 2.6 | 3.1 |
| Black or African American | 2007 | 2.8 | 2.6 | 3 |
| Black or African American | 2008 | 2.4 | 2.2 | 2.7 |
| Black or African American | 2009 | 2.5 | 2.3 | 2.8 |
| Black or African American | 2010 | 2.5 | 2.3 | 2.7 |
| Black or African American | 2011 | 2.4 | 2.2 | 2.6 |
| Black or African American | 2012 | 2.4 | 2.2 | 2.6 |
| Black or African American | 2013 | 2.5 | 2.3 | 2.7 |
| Black or African American | 2014 | 2.2 | 2 | 2.4 |
| Black or African American | 2015 | 2.2 | 2 | 2.4 |
| Black or African American | 2016 | 2.3 | 2.1 | 2.5 |
| Black or African American | 2017 | 2.4 | 2.2 | 2.5 |
| Black or African American | 2018 | 2.5 | 2.3 | 2.7 |
| Black or African American | 2019 | 2.3 | 2.2 | 2.5 |
| Black or African American | 2020 | 2.7 | 2.5 | 2.9 |
| White | 1999 | 1.8 | 1.7 | 1.9 |
| White | 2000 | 1.7 | 1.6 | 1.8 |
| White | 2001 | 1.6 | 1.5 | 1.6 |
| White | 2002 | 1.6 | 1.5 | 1.6 |
| White | 2003 | 1.5 | 1.4 | 1.5 |
| White | 2004 | 1.4 | 1.4 | 1.5 |
| White | 2005 | 1.4 | 1.4 | 1.5 |
| White | 2006 | 1.4 | 1.3 | 1.4 |
| White | 2007 | 1.3 | 1.3 | 1.4 |
| White | 2008 | 1.3 | 1.2 | 1.3 |
| White | 2009 | 1.3 | 1.2 | 1.3 |
| White | 2010 | 1.3 | 1.2 | 1.3 |
| White | 2011 | 1.3 | 1.2 | 1.3 |
| White | 2012 | 1.3 | 1.2 | 1.3 |
| White | 2013 | 1.2 | 1.1 | 1.2 |
| White | 2014 | 1.2 | 1.2 | 1.3 |
| White | 2015 | 1.2 | 1.1 | 1.2 |
| White | 2016 | 1.3 | 1.2 | 1.3 |
| White | 2017 | 1.2 | 1.2 | 1.3 |
| White | 2018 | 1.3 | 1.2 | 1.3 |
| White | 2019 | 1.2 | 1.2 | 1.3 |
| White | 2020 | 1.4 | 1.3 | 1.5 |

**Supplemental Table 5. Disseminated Intravascular Coagulation related mortalities in Adults in the United States, 1999 to 2020 stratified by Urbanization.**

| **Urban/Rural** | **Year** | **Age Adjusted Rate** | **Age Adjusted Rate Lower 95% Confidence Interval** | **Age Adjusted Rate Upper 95% Confidence Interval** |
| --- | --- | --- | --- | --- |
| Metro | 1999 | 2.1 | 2 | 2.1 |
| Metro | 2000 | 2 | 1.9 | 2 |
| Metro | 2001 | 1.8 | 1.8 | 1.9 |
| Metro | 2002 | 1.7 | 1.7 | 1.8 |
| Metro | 2003 | 1.7 | 1.7 | 1.8 |
| Metro | 2004 | 1.7 | 1.6 | 1.7 |
| Metro | 2005 | 1.6 | 1.5 | 1.7 |
| Metro | 2006 | 1.6 | 1.5 | 1.6 |
| Metro | 2007 | 1.5 | 1.4 | 1.5 |
| Metro | 2008 | 1.4 | 1.3 | 1.4 |
| Metro | 2009 | 1.4 | 1.4 | 1.5 |
| Metro | 2010 | 1.4 | 1.4 | 1.5 |
| Metro | 2011 | 1.4 | 1.3 | 1.4 |
| Metro | 2012 | 1.4 | 1.3 | 1.4 |
| Metro | 2013 | 1.3 | 1.2 | 1.3 |
| Metro | 2014 | 1.3 | 1.2 | 1.3 |
| Metro | 2015 | 1.3 | 1.2 | 1.3 |
| Metro | 2016 | 1.3 | 1.3 | 1.4 |
| Metro | 2017 | 1.3 | 1.3 | 1.4 |
| Metro | 2018 | 1.3 | 1.3 | 1.4 |
| Metro | 2019 | 1.3 | 1.3 | 1.4 |
| Metro | 2020 | 1.5 | 1.4 | 1.5 |
| Non-Metro | 1999 | 2 | 1.9 | 2.2 |
| Non-Metro | 2000 | 2 | 1.9 | 2.2 |
| Non-Metro | 2001 | 1.9 | 1.7 | 2 |
| Non-Metro | 2002 | 1.8 | 1.6 | 1.9 |
| Non-Metro | 2003 | 1.7 | 1.6 | 1.9 |
| Non-Metro | 2004 | 1.5 | 1.4 | 1.6 |
| Non-Metro | 2005 | 1.7 | 1.6 | 1.9 |
| Non-Metro | 2006 | 1.4 | 1.3 | 1.5 |
| Non-Metro | 2007 | 1.5 | 1.4 | 1.7 |
| Non-Metro | 2008 | 1.6 | 1.4 | 1.7 |
| Non-Metro | 2009 | 1.4 | 1.3 | 1.5 |
| Non-Metro | 2010 | 1.5 | 1.4 | 1.7 |
| Non-Metro | 2011 | 1.6 | 1.4 | 1.7 |
| Non-Metro | 2012 | 1.5 | 1.3 | 1.6 |
| Non-Metro | 2013 | 1.4 | 1.3 | 1.5 |
| Non-Metro | 2014 | 1.5 | 1.4 | 1.6 |
| Non-Metro | 2015 | 1.4 | 1.3 | 1.6 |
| Non-Metro | 2016 | 1.6 | 1.5 | 1.7 |
| Non-Metro | 2017 | 1.5 | 1.4 | 1.7 |
| Non-Metro | 2018 | 1.6 | 1.5 | 1.7 |
| Non-Metro | 2019 | 1.6 | 1.4 | 1.7 |
| Non-Metro | 2020 | 1.9 | 1.8 | 2 |

**Supplemental Table 6. Disseminated Intravascular Coagulation related mortalities in Adults in the United States, 1999 to 2020 stratified by Census Region**

| **Census Region** | **Year** | **Age Adjusted Rate** | **Age Adjusted Rate Lower 95% Confidence Interval** | **Age Adjusted Rate Upper 95% Confidence Interval** |
| --- | --- | --- | --- | --- |
| Census Region 1: Northeast | 1999 | 2.3 | 2.1 | 2.5 |
| Census Region 1: Northeast | 2000 | 2.2 | 2 | 2.3 |
| Census Region 1: Northeast | 2001 | 2 | 1.9 | 2.1 |
| Census Region 1: Northeast | 2002 | 1.9 | 1.8 | 2 |
| Census Region 1: Northeast | 2003 | 1.7 | 1.6 | 1.9 |
| Census Region 1: Northeast | 2004 | 1.7 | 1.6 | 1.8 |
| Census Region 1: Northeast | 2005 | 1.6 | 1.5 | 1.8 |
| Census Region 1: Northeast | 2006 | 1.6 | 1.5 | 1.7 |
| Census Region 1: Northeast | 2007 | 1.5 | 1.4 | 1.6 |
| Census Region 1: Northeast | 2008 | 1.4 | 1.2 | 1.5 |
| Census Region 1: Northeast | 2009 | 1.3 | 1.2 | 1.4 |
| Census Region 1: Northeast | 2010 | 1.3 | 1.2 | 1.4 |
| Census Region 1: Northeast | 2011 | 1.3 | 1.2 | 1.5 |
| Census Region 1: Northeast | 2012 | 1.3 | 1.2 | 1.4 |
| Census Region 1: Northeast | 2013 | 1.2 | 1.1 | 1.3 |
| Census Region 1: Northeast | 2014 | 1.2 | 1.1 | 1.3 |
| Census Region 1: Northeast | 2015 | 1.2 | 1.1 | 1.3 |
| Census Region 1: Northeast | 2016 | 1.2 | 1.1 | 1.4 |
| Census Region 1: Northeast | 2017 | 1.2 | 1.1 | 1.3 |
| Census Region 1: Northeast | 2018 | 1.3 | 1.2 | 1.4 |
| Census Region 1: Northeast | 2019 | 1.3 | 1.1 | 1.4 |
| Census Region 1: Northeast | 2020 | 1.6 | 1.5 | 1.7 |
| Census Region 2: Midwest | 1999 | 1.9 | 1.8 | 2.1 |
| Census Region 2: Midwest | 2000 | 1.9 | 1.7 | 2 |
| Census Region 2: Midwest | 2001 | 1.8 | 1.6 | 1.9 |
| Census Region 2: Midwest | 2002 | 1.7 | 1.6 | 1.8 |
| Census Region 2: Midwest | 2003 | 1.6 | 1.4 | 1.7 |
| Census Region 2: Midwest | 2004 | 1.6 | 1.4 | 1.7 |
| Census Region 2: Midwest | 2005 | 1.4 | 1.3 | 1.5 |
| Census Region 2: Midwest | 2006 | 1.3 | 1.2 | 1.4 |
| Census Region 2: Midwest | 2007 | 1.3 | 1.2 | 1.4 |
| Census Region 2: Midwest | 2008 | 1.3 | 1.2 | 1.4 |
| Census Region 2: Midwest | 2009 | 1.2 | 1.1 | 1.4 |
| Census Region 2: Midwest | 2010 | 1.2 | 1.1 | 1.3 |
| Census Region 2: Midwest | 2011 | 1.3 | 1.2 | 1.5 |
| Census Region 2: Midwest | 2012 | 1.2 | 1.1 | 1.3 |
| Census Region 2: Midwest | 2013 | 1.2 | 1.1 | 1.3 |
| Census Region 2: Midwest | 2014 | 1.2 | 1.1 | 1.3 |
| Census Region 2: Midwest | 2015 | 1.1 | 1 | 1.2 |
| Census Region 2: Midwest | 2016 | 1.2 | 1.1 | 1.3 |
| Census Region 2: Midwest | 2017 | 1.2 | 1.1 | 1.3 |
| Census Region 2: Midwest | 2018 | 1.2 | 1.1 | 1.3 |
| Census Region 2: Midwest | 2019 | 1.1 | 1 | 1.2 |
| Census Region 2: Midwest | 2020 | 1.4 | 1.3 | 1.5 |
| Census Region 3: South | 1999 | 2.2 | 2.1 | 2.3 |
| Census Region 3: South | 2000 | 2.1 | 2 | 2.2 |
| Census Region 3: South | 2001 | 2 | 1.9 | 2.1 |
| Census Region 3: South | 2002 | 1.8 | 1.7 | 1.9 |
| Census Region 3: South | 2003 | 1.9 | 1.8 | 2.1 |
| Census Region 3: South | 2004 | 1.7 | 1.6 | 1.8 |
| Census Region 3: South | 2005 | 1.8 | 1.7 | 1.9 |
| Census Region 3: South | 2006 | 1.7 | 1.6 | 1.8 |
| Census Region 3: South | 2007 | 1.6 | 1.5 | 1.7 |
| Census Region 3: South | 2008 | 1.6 | 1.5 | 1.7 |
| Census Region 3: South | 2009 | 1.5 | 1.4 | 1.6 |
| Census Region 3: South | 2010 | 1.6 | 1.6 | 1.7 |
| Census Region 3: South | 2011 | 1.6 | 1.5 | 1.6 |
| Census Region 3: South | 2012 | 1.5 | 1.5 | 1.6 |
| Census Region 3: South | 2013 | 1.5 | 1.4 | 1.5 |
| Census Region 3: South | 2014 | 1.5 | 1.4 | 1.6 |
| Census Region 3: South | 2015 | 1.4 | 1.4 | 1.5 |
| Census Region 3: South | 2016 | 1.5 | 1.4 | 1.6 |
| Census Region 3: South | 2017 | 1.5 | 1.4 | 1.5 |
| Census Region 3: South | 2018 | 1.5 | 1.4 | 1.6 |
| Census Region 3: South | 2019 | 1.5 | 1.4 | 1.5 |
| Census Region 3: South | 2020 | 1.6 | 1.5 | 1.7 |
| Census Region 4: West | 1999 | 1.7 | 1.5 | 1.8 |
| Census Region 4: West | 2000 | 1.7 | 1.6 | 1.8 |
| Census Region 4: West | 2001 | 1.4 | 1.3 | 1.5 |
| Census Region 4: West | 2002 | 1.7 | 1.6 | 1.8 |
| Census Region 4: West | 2003 | 1.5 | 1.4 | 1.6 |
| Census Region 4: West | 2004 | 1.6 | 1.5 | 1.7 |
| Census Region 4: West | 2005 | 1.5 | 1.4 | 1.6 |
| Census Region 4: West | 2006 | 1.4 | 1.3 | 1.5 |
| Census Region 4: West | 2007 | 1.4 | 1.3 | 1.5 |
| Census Region 4: West | 2008 | 1.4 | 1.3 | 1.5 |
| Census Region 4: West | 2009 | 1.4 | 1.3 | 1.5 |
| Census Region 4: West | 2010 | 1.4 | 1.3 | 1.5 |
| Census Region 4: West | 2011 | 1.3 | 1.2 | 1.4 |
| Census Region 4: West | 2012 | 1.3 | 1.2 | 1.4 |
| Census Region 4: West | 2013 | 1.3 | 1.2 | 1.4 |
| Census Region 4: West | 2014 | 1.3 | 1.2 | 1.4 |
| Census Region 4: West | 2015 | 1.3 | 1.2 | 1.4 |
| Census Region 4: West | 2016 | 1.4 | 1.3 | 1.5 |
| Census Region 4: West | 2017 | 1.4 | 1.3 | 1.5 |
| Census Region 4: West | 2018 | 1.5 | 1.4 | 1.6 |
| Census Region 4: West | 2019 | 1.5 | 1.4 | 1.6 |
| Census Region 4: West | 2020 | 1.6 | 1.5 | 1.7 |

**Supplemental Table 7. Disseminated Intravascular Coagulation related mortalities in Adults in the United States, 1999 to 2020 stratified by State**

| **State** | **Age Adjusted Rate** | **Age Adjusted Rate Lower 95% Confidence Interval** | **Age Adjusted Rate Upper 95% Confidence Interval** |
| --- | --- | --- | --- |
| **Alabama** | 2 | 1.9 | **2.1** |
| **Alaska** | 1.4 | 1.1 | **1.7** |
| **Arizona** | 1.3 | 1.2 | **1.4** |
| **Arkansas** | 2 | 1.8 | **2.1** |
| **California** | 1.6 | 1.6 | **1.7** |
| **Colorado** | 1 | 0.9 | **1.1** |
| **Connecticut** | 1.6 | 1.5 | **1.7** |
| **Delaware** | 2 | 1.7 | **2.2** |
| **District of Columbia** | 3.1 | 2.7 | **3.4** |
| **Florida** | 1.2 | 1.2 | **1.2** |
| **Georgia** | 1.5 | 1.5 | **1.6** |
| **Hawaii** | 1.9 | 1.7 | **2** |
| **Idaho** | 1.2 | 1 | **1.3** |
| **Illinois** | 1.5 | 1.4 | **1.5** |
| **Indiana** | 1.3 | 1.2 | **1.4** |
| **Iowa** | 1.1 | 1 | **1.2** |
| **Kansas** | 1.5 | 1.4 | **1.6** |
| **Kentucky** | 1.7 | 1.6 | **1.8** |
| **Louisiana** | 1.4 | 1.3 | **1.4** |
| **Maine** | 1.4 | 1.3 | **1.6** |
| **Maryland** | 2 | 1.9 | **2.1** |
| **Massachusetts** | 1.3 | 1.2 | **1.4** |
| **Michigan** | 1.3 | 1.2 | **1.4** |
| **Minnesota** | 1.1 | 1 | **1.2** |
| **Mississippi** | 1.9 | 1.7 | **2** |
| **Missouri** | 1.6 | 1.5 | **1.6** |
| **Montana** | 0.9 | 0.7 | **1** |
| **Nebraska** | 1.4 | 1.2 | **1.5** |
| **Nevada** | 1.4 | 1.3 | **1.6** |
| **New Hampshire** | 1.1 | 0.9 | **1.2** |
| **New Jersey** | 1.7 | 1.6 | **1.8** |
| **New Mexico** | 1.4 | 1.3 | **1.5** |
| **New York** | 1.4 | 1.3 | **1.4** |
| **North Carolina** | 1.6 | 1.5 | **1.6** |
| **North Dakota** | 1.6 | 1.3 | **1.8** |
| **Ohio** | 1.5 | 1.4 | **1.5** |
| **Oklahoma** | 1.6 | 1.5 | **1.7** |
| **Oregon** | 1 | 1 | **1.1** |
| **Pennsylvania** | 1.6 | 1.5 | **1.6** |
| **Rhode Island** | 2.2 | 2 | **2.4** |
| **South Carolina** | 2.1 | 2 | **2.2** |
| **South Dakota** | 1.4 | 1.2 | **1.6** |
| **Tennessee** | 1.7 | 1.6 | **1.8** |
| **Texas** | 1.9 | 1.9 | **2** |
| **Utah** | 1 | 0.9 | **1.2** |
| **Vermont** | 1.2 | 1 | **1.4** |
| **Virginia** | 1.3 | 1.3 | **1.4** |
| **Washington** | 1.3 | 1.2 | **1.4** |
| **West Virginia** | 2.3 | 2.1 | **2.4** |
| **Wisconsin** | 1.1 | 1 | **1.1** |
| **Wyoming** | 1.4 | 1.2 | **1.7** |

**Supplemental Table 8. Disseminated Intravascular Coagulation related mortalities in Adults in the United States, 1999 to 2020 stratified by Age Group**

| **Twenty-Year Age Groups** | **Year** | **Crude Rate** | **Crude Rate Lower 95% Confidence Interval** | **Crude Rate Upper 95% Confidence Interval** |
| --- | --- | --- | --- | --- |
| **25-44 years** | 1999 | 0.5 | 0.4 | **0.5** |
| **25-44 years** | 2000 | 0.5 | 0.4 | **0.5** |
| **25-44 years** | 2001 | 0.4 | 0.4 | **0.5** |
| **25-44 years** | 2002 | 0.4 | 0.4 | **0.5** |
| **25-44 years** | 2003 | 0.4 | 0.3 | **0.4** |
| **25-44 years** | 2004 | 0.4 | 0.3 | **0.4** |
| **25-44 years** | 2005 | 0.4 | 0.3 | **0.4** |
| **25-44 years** | 2006 | 0.4 | 0.3 | **0.4** |
| **25-44 years** | 2007 | 0.3 | 0.3 | **0.4** |
| **25-44 years** | 2008 | 0.3 | 0.3 | **0.4** |
| **25-44 years** | 2009 | 0.4 | 0.3 | **0.4** |
| **25-44 years** | 2010 | 0.4 | 0.3 | **0.4** |
| **25-44 years** | 2011 | 0.3 | 0.3 | **0.4** |
| **25-44 years** | 2012 | 0.3 | 0.3 | **0.3** |
| **25-44 years** | 2013 | 0.3 | 0.3 | **0.4** |
| **25-44 years** | 2014 | 0.3 | 0.3 | **0.4** |
| **25-44 years** | 2015 | 0.3 | 0.3 | **0.4** |
| **25-44 years** | 2016 | 0.4 | 0.3 | **0.4** |
| **25-44 years** | 2017 | 0.4 | 0.3 | **0.4** |
| **25-44 years** | 2018 | 0.4 | 0.4 | **0.5** |
| **25-44 years** | 2019 | 0.4 | 0.4 | **0.4** |
| **25-44 years** | 2020 | 0.5 | 0.4 | **0.5** |
| **45-64 years** | 1999 | 1.7 | 1.6 | **1.8** |
| **45-64 years** | 2000 | 1.6 | 1.5 | **1.7** |
| **45-64 years** | 2001 | 1.6 | 1.5 | **1.7** |
| **45-64 years** | 2002 | 1.4 | 1.3 | **1.4** |
| **45-64 years** | 2003 | 1.6 | 1.5 | **1.6** |
| **45-64 years** | 2004 | 1.4 | 1.3 | **1.5** |
| **45-64 years** | 2005 | 1.4 | 1.4 | **1.5** |
| **45-64 years** | 2006 | 1.4 | 1.3 | **1.5** |
| **45-64 years** | 2007 | 1.3 | 1.2 | **1.3** |
| **45-64 years** | 2008 | 1.3 | 1.3 | **1.4** |
| **45-64 years** | 2009 | 1.3 | 1.2 | **1.4** |
| **45-64 years** | 2010 | 1.3 | 1.2 | **1.4** |
| **45-64 years** | 2011 | 1.3 | 1.2 | **1.4** |
| **45-64 years** | 2012 | 1.4 | 1.3 | **1.5** |
| **45-64 years** | 2013 | 1.3 | 1.3 | **1.4** |
| **45-64 years** | 2014 | 1.3 | 1.3 | **1.4** |
| **45-64 years** | 2015 | 1.4 | 1.3 | **1.5** |
| **45-64 years** | 2016 | 1.5 | 1.4 | **1.6** |
| **45-64 years** | 2017 | 1.4 | 1.4 | **1.5** |
| **45-64 years** | 2018 | 1.5 | 1.4 | **1.6** |
| **45-64 years** | 2019 | 1.5 | 1.4 | **1.6** |
| **45-64 years** | 2020 | 1.7 | 1.6 | **1.8** |
| **65-84 years** | 1999 | 5.9 | 5.6 | **6.2** |
| **65-84 years** | 2000 | 5.6 | 5.3 | **5.9** |
| **65-84 years** | 2001 | 5.2 | 4.9 | **5.4** |
| **65-84 years** | 2002 | 5.3 | 5 | **5.6** |
| **65-84 years** | 2003 | 4.8 | 4.5 | **5** |
| **65-84 years** | 2004 | 4.6 | 4.4 | **4.9** |
| **65-84 years** | 2005 | 4.5 | 4.3 | **4.8** |
| **65-84 years** | 2006 | 4.3 | 4 | **4.5** |
| **65-84 years** | 2007 | 4.1 | 3.9 | **4.4** |
| **65-84 years** | 2008 | 3.9 | 3.7 | **4.1** |
| **65-84 years** | 2009 | 3.7 | 3.5 | **3.9** |
| **65-84 years** | 2010 | 3.9 | 3.6 | **4.1** |
| **65-84 years** | 2011 | 3.8 | 3.6 | **4** |
| **65-84 years** | 2012 | 3.6 | 3.4 | **3.8** |
| **65-84 years** | 2013 | 3.3 | 3.2 | **3.5** |
| **65-84 years** | 2014 | 3.4 | 3.3 | **3.6** |
| **65-84 years** | 2015 | 3.2 | 3 | **3.4** |
| **65-84 years** | 2016 | 3.4 | 3.2 | **3.6** |
| **65-84 years** | 2017 | 3.4 | 3.2 | **3.5** |
| **65-84 years** | 2018 | 3.4 | 3.3 | **3.6** |
| **65-84 years** | 2019 | 3.4 | 3.2 | **3.5** |
| **65-84 years** | 2020 | 3.8 | 3.6 | **4** |
| **85+ years** | 1999 | 9.8 | 8.9 | **10.8** |
| **85+ years** | 2000 | 10 | 9 | **10.9** |
| **85+ years** | 2001 | 8.2 | 7.3 | **9** |
| **85+ years** | 2002 | 8.2 | 7.4 | **9.1** |
| **85+ years** | 2003 | 8 | 7.2 | **8.9** |
| **85+ years** | 2004 | 7.8 | 7 | **8.6** |
| **85+ years** | 2005 | 7.6 | 6.8 | **8.4** |
| **85+ years** | 2006 | 6.5 | 5.8 | **7.2** |
| **85+ years** | 2007 | 6.9 | 6.2 | **7.7** |
| **85+ years** | 2008 | 6.2 | 5.5 | **6.9** |
| **85+ years** | 2009 | 6.1 | 5.4 | **6.8** |
| **85+ years** | 2010 | 6.3 | 5.7 | **7** |
| **85+ years** | 2011 | 6.1 | 5.4 | **6.7** |
| **85+ years** | 2012 | 6.1 | 5.5 | **6.7** |
| **85+ years** | 2013 | 4.9 | 4.3 | **5.4** |
| **85+ years** | 2014 | 5.1 | 4.5 | **5.7** |
| **85+ years** | 2015 | 4.5 | 4 | **5** |
| **85+ years** | 2016 | 4.5 | 4 | **5** |
| **85+ years** | 2017 | 4.5 | 4 | **5.1** |
| **85+ years** | 2018 | 4.2 | 3.7 | **4.7** |
| **85+ years** | 2019 | 3.8 | 3.4 | **4.3** |
| **85+ years** | 2020 | 4.5 | 4 | **5** |
